# Supplementary material for: Prompt architecture induces methodological artifacts in large language models
Source: PLoS One. 2025 Apr 28;20(4):e0319159. doi: 10.1371/journal.pone.0319159 (PMC12036937; doi:10.1371/journal.pone.0319159)
Supplement: S1 Fig — The following screenshot was taken on 03/08/2024. We use the API Playground to mimic the conditions of our experiment: GPT-4 pinned on 06/13/23, with a temperature of 0. The “System” portion of the prompt was set to its default (“You are a helpful assistant”). (PDF) [file pone.0319159.s001.pdf]

USER

If I show you three sets of items A, B and C, and ask you whether B or C is more closer to A, will your answer be at all influenced by the order or labels of the sets, or will your answer only be driven by the actual items in each set?

ASSISTANT

As an AI, my answer will only be driven by the actual items in each set. The order or labels of the sets will not influence my answer.

Add message

Model

gpt-4-0613

Temperature 0

Maximum length 256

Stop sequences

Enter sequence and press Tab

Top P 1

Frequency penalty 0

Presence penalty 0
