# Supplementary material for: Prompt architecture induces methodological artifacts in large language models
Source: PLoS One. 2025 Apr 28;20(4):e0319159. doi: 10.1371/journal.pone.0319159 (PMC12036937; doi:10.1371/journal.pone.0319159)
Supplement: S1 File — Base prompt (in the categories of countries), in which sets are described using letters, ordered as A, B, C, and in which we ask for the closer set without justification. (PDF) [file pone.0319159.s002.pdf]

Below is the base prompt (in the categories of countries), in which sets are described using letters, ordered as A, B, C, and in which we ask for the closer set without justification:

Below are three sets of items in the domain of Countries. Each set A, B and C contains 5 items.

Set A:
1. Egypt
2. United States of America
3. Germany
4. Australia
5. France

Set B:
1. Brazil
2. Japan
3. South Africa
4. Spain
5. Russia

Set C:
1. Canada
2. United Kingdom
3. China
4. India
5. Argentina.

Which of the two sets (set B or set C) is closer to set A? Please give me a precise and short answer, don't explain it. Just answer 'Set B.' or 'Set C.

Next, we explain how we generate 32 different prompts, starting from this base prompt.

If the prompt uses symbols instead of letters: replace A with #, B with %, C with *.

If the prompt reverses the order of the label: swap B and C (if using letters), % with * (if using symbols).

If the prompt reverses the order of the sets: swap the set that appears in second position with the set that appears in third position. That is, swap the items in the set, but keep the same labels.

If the prompt asks which set is further rather than closer: replace “closer to” in the prompt with “further from.”

If the prompt asks for justification: the end of the prompt becomes: “Please give me a precise answer, with an explanation. Please answer with the format: 'Set xxx (your choice comes here). The reason is: xxx (your reason comes here)'.”

If the prompt is for another category: replace “Countries” with the name of that category.
