# Supplementary material for: Prompt architecture induces methodological artifacts in large language models
Source: PLoS One. 2025 Apr 28;20(4):e0319159. doi: 10.1371/journal.pone.0319159 (PMC12036937; doi:10.1371/journal.pone.0319159)
Supplement: S2 File — (PDF) [file pone.0319159.s003.pdf]

We randomly generate 30 triplets of sets of items in six categories (countries, professions, hobbies, meals, symptoms, animals) by conducting the following procedure 10 times (each replication generates three triplets per category). We first ask GPT-4 (with a temperature of 0) to generate 15 items in that category. Then, we generate three triplets of sets of items, where each triplet is obtained by randomly drawing (without replacement) five items for each set. See the data files on Researchbox.org for a full list of items and prompts.
