# Supplementary material for: Prompt architecture induces methodological artifacts in large language models
Source: PLoS One. 2025 Apr 28;20(4):e0319159. doi: 10.1371/journal.pone.0319159 (PMC12036937; doi:10.1371/journal.pone.0319159)
Supplement: S3 File — (PDF) [file pone.0319159.s004.pdf]

We replicate our experiment using the exact same experimental design on Llama 3.1-8B (again with a temperature of 0).

Out of 5,760 observations, Llama 3.1 failed to complete the task 57 times, a failure rate of 1%. We removed these observations to yield our final dataset of 5,703 observations.

Response Order and Label Bias. Given full randomization, an unbiased responder should select the first option 50% of the time and any specific label 50% of the time. Instead, we find evidence that Llama 3.1 is prone to response-order bias and label bias. On average, across all observations, Llama 3.1 selected the first option in 58.14% of the cases (p <.001) and selected B over C in 76.70% of the cases (p <.001). In contrast to GPT-4, we find that label bias is not reduced when using symbols: the symbol % is chosen over the symbol * in 76.73% of the cases (versus 76.70% of 'B' for letters). Interestingly, Llama 3.1 preferred the same symbol (% over *) as GPT-4.

Interaction of Architectural Elements. First, we examine how response-order and label bias interact with each other. As with GPT-4, we find that response-order bias is much stronger when using symbols (which are uncommon labels) than when using letters (which are common labels). Llama 3.1 selected the first option in 50.74% of the cases that were labeled with letters and 65.40% of the cases that were labeled with symbols.

We next examine the two additional architectural elements of justification and framing and test whether they impact response-order or label bias (letters and symbols). We find that asking Llama 3.1 for justification slightly (but non-sognificantly) reduces response-order bias (from the first option being selected in 59.34 % of cases to 56.93% of cases, p = .065) but exacerbated lettered-label bias (from 'B' being selected in 64.10% of cases to 89.81% of cases, p < .001) and symbol-label bias (from '%' being selected in 69.31% of cases to 84.16% of cases, p < .001).

We also find that framing interacts with both response order and label bias. Specifically, we find that both response-order and lettered-label bias are stronger when Llama 3.1 is asked which set is closer to a given stimuli than when it is asked which set is further (from the first option being selected in 77.22 % of cases with closer framing to 39.44% of cases with further framing, and from option 'B' being selected in 80.27% of cases with closer framing to 73.26% of cases with further framing) but that label bias with symbols is stronger when Llama 3.1 is asked which set is further (from option '%' being selected in 91.04% of cases with further framing to 62.40% of cases with closer framing, ps < .001).
