# Supplementary material for: Prompt architecture induces methodological artifacts in large language models
Source: PLoS One. 2025 Apr 28;20(4):e0319159. doi: 10.1371/journal.pone.0319159 (PMC12036937; doi:10.1371/journal.pone.0319159)
Supplement: S4 File — (PDF) [file pone.0319159.s005.pdf]

Although older versions of GPT such as GPT-3 are likely to be phased out soon, there is still value in testing older versions of GPT for potential biases to examine whether biases are increasing, decreasing, or changing in more complicated manners across versions of GPT. For example, Kosinski (2023) finds that versions of GPT have increasing Theory of Mind abilities (1). If we find that GPT-4 is less biased than GPT-3, this would be encouraging evidence that biases are likely to be increasingly small as GPT evolves. Accordingly, we replicate our experiment using the exact same experimental design on GPT-3 (we use the davinci 3 model, again with a temperature of 0).

We again start by testing for potential bias in favor of the first option. As reported in the main text, response-order bias appears to be less severe in GPT-3 (first option selected in 53.16% of the cases) compared to GPT-4 (first option selected in 63.21% of the cases).

Breaking down the results per condition, the pattern of results is different from the one observed with GPT-4 and reported in the main text. Here, the bias appears to be severe in some conditions (e.g., when asking which set is closer, with 74.41% of cases in which the first option was selected), but it is actually reversed in other conditions. For example, when asking which set is further, the first option listed is selected in only 31.91% of the cases, which is significantly lower than 50% ($p < .001$).

Next, we look again at potential bias in favor of B over C in cases in which letters are used as labels. While B is chosen in 64.10% of the cases (significantly above 50%, $p < .001$), there is considerable heterogeneity across conditions, pictured in the graphs below.

Finally, we explore bias for symbols. We find that % is chosen over * in 77.15% of the cases. This bias in favor of % over * is quite robust across conditions and particularly strong when % is the first option listed and when asking which set is closer (100% of the time, GPT-3 selects %).

In sum, it seems hard to find a pattern in the way prompt architecture effects are evolving across versions of GPT, and we do not find any evidence that these methodological biases are being systematically reduced in newer versions of GPT. This underscores the unpredictability of how and when GPT will be biased by the methodological context of the study. Using LLMs as a source of data mirrors human research to the extent that anticipating the numerous ways human participants might be biased is similarly challenging.

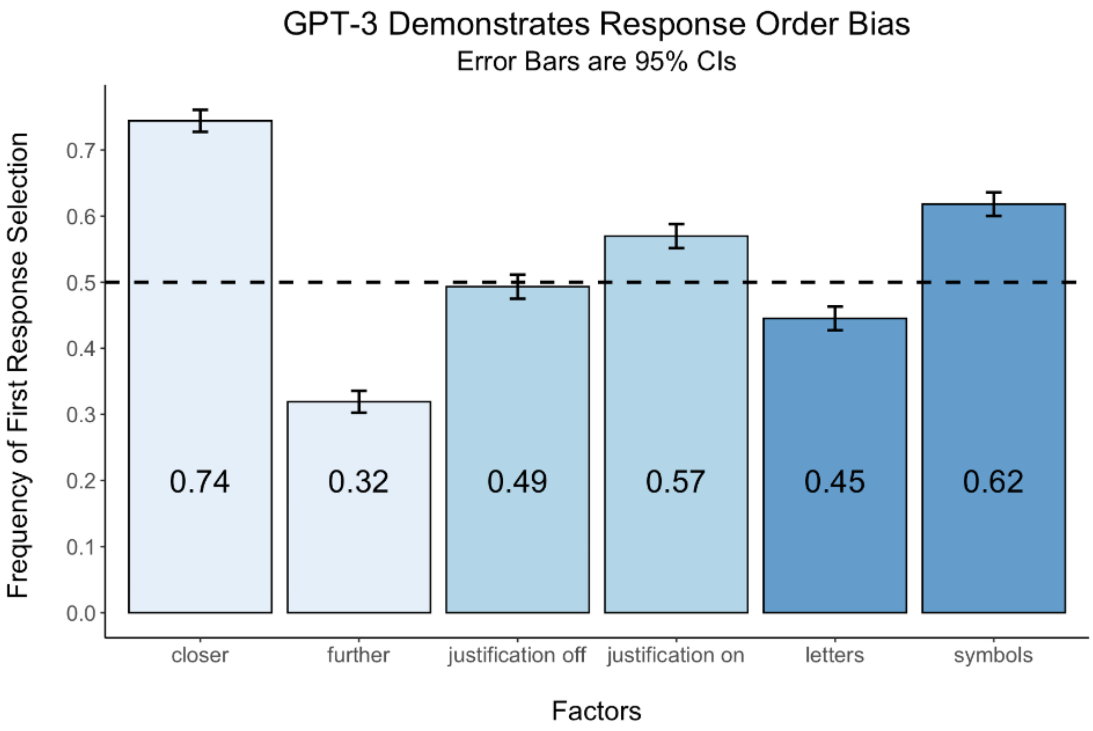
GPT-3 Demonstrates Response Order Bias
Error Bars are 95% CIs
Frequency of First Response Selection
0.0
0.1
0.2
0.3
0.4
0.5
0.6
0.7
0.74
0.32
0.49
0.57
0.45
0.62
closer
further
justification off
justification on
letters
symbols
Factors

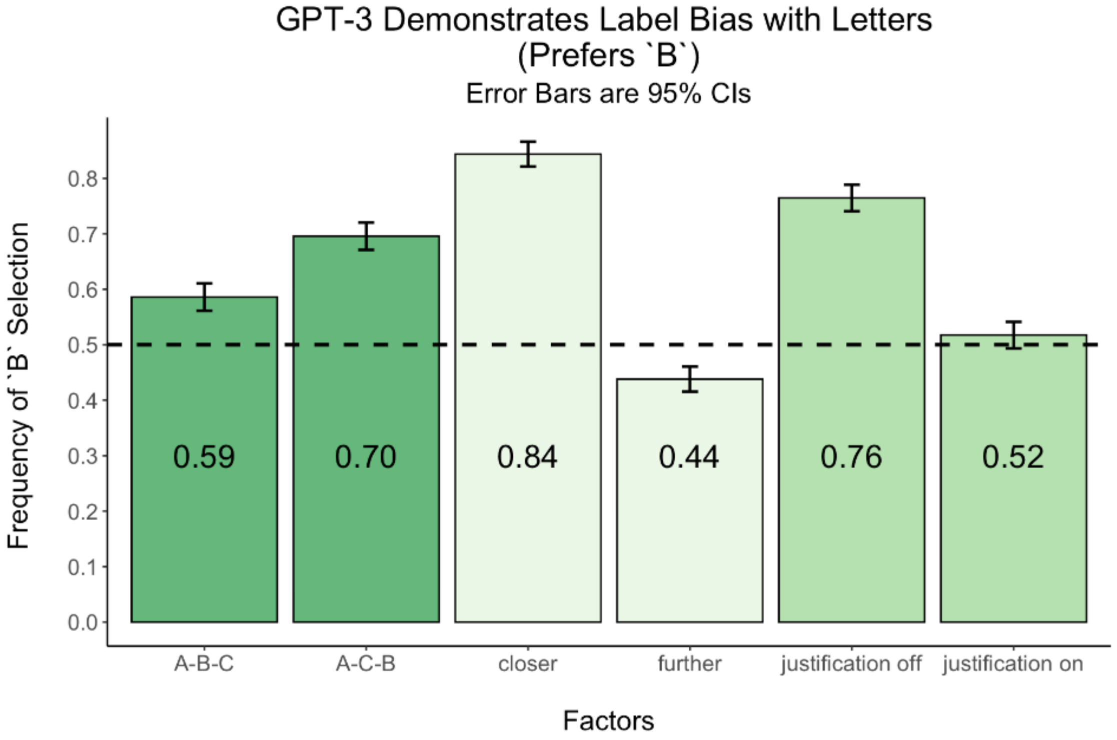
GPT-3 Demonstrates Label Bias with Letters
(Prefers `B`)
Error Bars are 95% CIs
Frequency of `B` Selection
0.0
0.1
0.2
0.3
0.4
0.5
0.6
0.7
0.8
0.59
0.70
0.84
0.44
0.76
0.52
A-B-C
A-C-B
closer
further
justification off
justification on
Factors

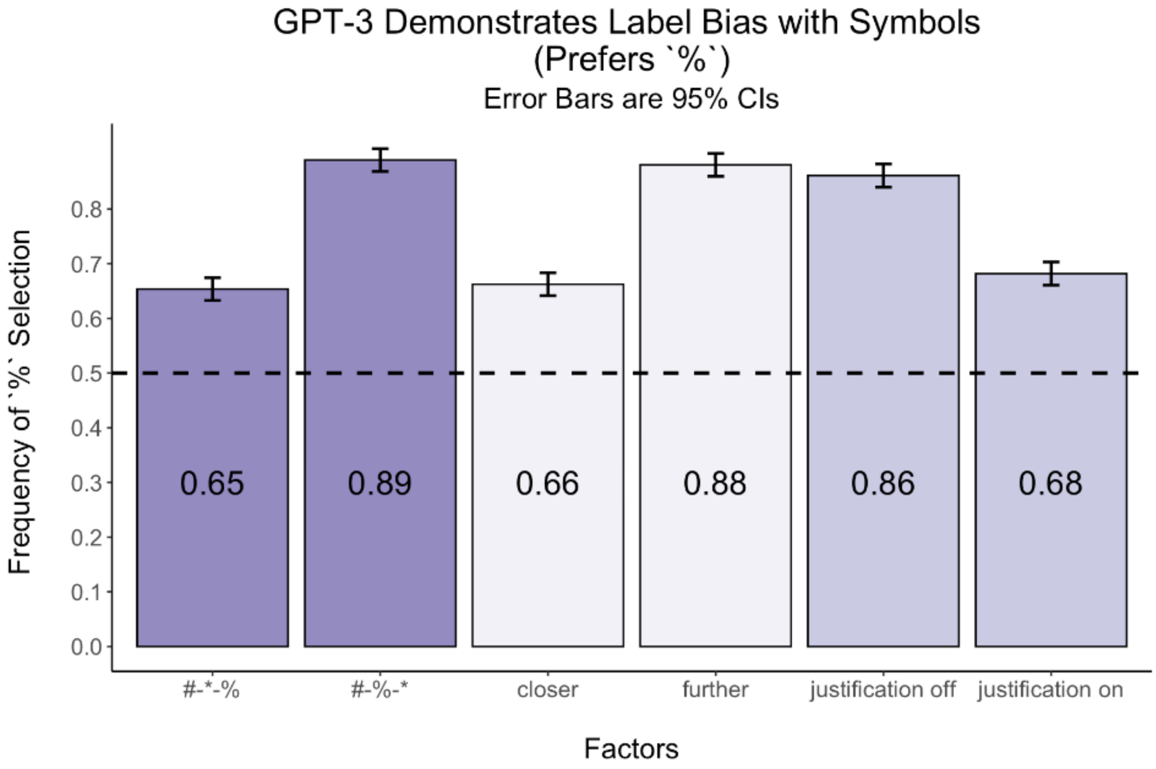

1. Kosinski M. Theory of Mind May Have Spontaneously Emerged in Large Language Models [Internet]. arXiv; 2023 [cited 2023 Jun 18]. Available from: http://arxiv.org/abs/2302.02083
