# Supplementary material for: Prompt architecture induces methodological artifacts in large language models
Source: PLoS One. 2025 Apr 28;20(4):e0319159. doi: 10.1371/journal.pone.0319159 (PMC12036937; doi:10.1371/journal.pone.0319159)
Supplement: S5 File — (PDF) [file pone.0319159.s006.pdf]

We replicate our experiment using the exact same experimental design on Llama 3.1 (again with a temperature of 0).

Out of 64,800 observations, Llama 3.1 never failed to complete the task; thus, our final dataset includes all 64,800 observations.

Response Order and Label Bias. On average, across all observations, we find response-order and label bias among these simpler, single-word items: Llama 3.1 selected the first option in 44.32% of the cases ($p <.001$) and selected response 'B' is 54.70% of cases ($p <.001$).

Intervention. Unlike GPT-4, response-order bias is not consistent across the Control, Chosen and Randomized conditions. Llama 3.1 selected the first option in 52.32% of the cases in the control condition ($p < .001$). However, Llama 3.1 selected the first option in only 37.75% of the time in the Randomized condition ($p < .001$) and only 42.89% of the time in the Chosen condition ($p < .001$). Thus, the condition did not reduce bias; instead, it exacerbated and flipped bias. The bias in favor of the label B over C, on the other hand, is again quite consistent across conditions: The frequency of B selection is 53.75% in the Control condition, 54.35% in the Randomized condition, 56.01% in the Chosen condition.
