# Supplementary material for: Prompt architecture induces methodological artifacts in large language models
Source: PLoS One. 2025 Apr 28;20(4):e0319159. doi: 10.1371/journal.pone.0319159 (PMC12036937; doi:10.1371/journal.pone.0319159)
Supplement: S6 File — (PDF) [file pone.0319159.s007.pdf]

In a separate study, we repeated the same design as Study 1 but switched to single items so there would be less ambiguity as how to compute similarity between items. We then used Word2Vec (Mikolov et al., 2017) embeddings to calculate the cosine similarity between item 1 and item 2 and between item 1 and item 3. From there, we create a proxy for accuracy by measuring whether GPT-4 selected the response consistent with the word embedding scores (e.g., selected the set with higher cosine similarity to item 1 if asked which item was closer).

We use our proxy for accuracy (calculated via cosine similarity) to quantify the extent to which task performance improves when using a simple aggregation strategy vs. the prompt someone might naturally employ without regard to prompt architecture. Specifically, we calculate the percentage of triplets where the majority response is considered correct based on our proxy, i.e., aggregating responses using a simple majority rule would lead to a correct answer, compared to the accuracy of all prompts with “closer” framing and A-B-C labeling (the likely default). We find that with aggregation, GPT-4 accurately responds for 67.22% of the triplets, an 11.36% increase compared to using the default prompt architecture (60.36%). These results provide preliminary evidence that aggregation improves GPT-4 performance, even with a noisy measure of word embeddings to approximate accuracy.

References

Mikolov, Tomas, et al. "Advances in pre-training distributed word representations." *arXiv preprint arXiv:1712.09405* (2017).
