# Supplementary material for: Prompt architecture induces methodological artifacts in large language models
Source: PLoS One. 2025 Apr 28;20(4):e0319159. doi: 10.1371/journal.pone.0319159 (PMC12036937; doi:10.1371/journal.pone.0319159)
Supplement: Attachment — Submitted filename: PONE-D-24-16087R1_comments.pdf [file pone.0319159.s009.pdf]

Line-by-line comments

38: Consider rephrasing “virtually unlimited memory and processing abilities” to avoid overstating LLMs computing power. While it is true that LLMs do not experience fatigue or require rest like humans, they are subject to memory constraints (e.g., character limits in prompts).

53: Applying the choice architecture framework is very appropriate. However, additional citations beyond the foundational work by Thaler and Sunstein (2009) would strengthen the paper. Possible sources include:

Johnson, E. J., Hershey, J., Meszaros, J., & Kunreuther, H. (1993). Framing, probability distortions, and insurance decisions. *Journal of Risk and Uncertainty 7*, 35–51. https://doi.org/10.1007/BF01065313

Mrkva, K., Posner, N. A., Reeck, C., & Johnson, E. J. (2021). Do nudges reduce disparities? Choice architecture compensates for low consumer knowledge. *Journal of Marketing, 85*(4), 67–84. https://doi.org/10.1177/0022242921993186

Thaler, Richard H. and Sunstein, Cass R. and Balz, John P., Choice Architecture (April 2, 2010). http://doi.org/10.2139/ssrn.1583509

69: The initial clarification that “justification” equates to “asking for reasons” is helpful. Afterward, maintaining consistent terminology by using justification aligns better with the choice architecture framework.

120: While this similarity task may be standard in psychology, the rationale for selecting it here is unclear. Could a more complex task (e.g., categorization) or simpler one (e.g., recognition) have provided additional insights relevant to the study?

126–141: The full-factorial design described here is both clever and effective. Presenting the information as a summary table could improve clarity. For instance:

**Bias**	**Example**
Order	A, B, C vs. B, A, C
Label	A, B, C vs. #, %, *
Framing	“Which is closer?” vs. “Which is farther?”
Justification	Justification request vs. none

147–148: The structured prompt used in the study is well-suited for research but may not reflect typical user behavior. Users often input shorthand, unstructured queries, such as:
“is set b or set c closer to set a: egypt us germany australia france. set b: brazil japan south africa spain. set c: canada uk china india argentina”

Has GPT-4's performance with less structured inputs been evaluated? While the authors note that GPT-4 will eventually be succeeded by future models, acknowledging that user prompts are often informal and variable could add practical depth to the analysis.

184–186:The figures are informative, but it might help if Figures B and C are grouped together, as they both illustrate label bias. Additionally, consider swapping the two framing bars in Figure A with the two label bars. This adjustment would align the visual organization with Figures B and C

192–205: The parallels between human and LLM decision-making are fascinating but could be misleading. The phrasing might suggest that LLMs have emotions or motivations. Emphasizing that these changes reflect *structural* patterns, not emotions, would clarify the distinction.

225: Consider replacing "irrationally" with "predictably," as these biases follow rational heuristics for efficiency. Both humans and LLMs are acting consistently based on context.

281: Including the percentage of failures would contextualize the impact of 489 errors. Additionally, the reported figure of 64,800 observations seems to include an extra "0."

358–365 & 367–376: These points are significant and belong in the methods section rather than the discussion, as they are not new findings but validation steps. The discussion should focus on interpreting results rather than supplying new information. Also, explicitly state that Word2Vec was used for word embeddings, as it is currently only specified in the supplementary materials.

376–378: Referring back to lines 109–111:
The recommendation to aggregate across different prompts is valuable, but it raises practical questions. Specifically:

> Is the final recommendation to use a full factorial search query? If so, this approach may be impractical for many users.
>
> If not a full factorial search, could the authors suggest a reasonable number of prompts that would sufficiently reduce bias?

Comments on Supplementary Material B:

There appears to be a typo:"If the prompt uses letters instead of symbols: replace A with #, B with %, C with ." It seems "letters" and "symbols" need to be swapped.

Additionally, the third technique described—swapping sets while maintaining labels—is a key aspect of prompt generation and should be referenced in the main text, not just the supplementary materials.
