# Supplementary material for: Prompt architecture induces methodological artifacts in large language models
Source: PLoS One. 2025 Apr 28;20(4):e0319159. doi: 10.1371/journal.pone.0319159 (PMC12036937; doi:10.1371/journal.pone.0319159)
Supplement: Attachment — Submitted filename: LLM_Research_Design_response_letter_final.docx [file pone.0319159.s010.docx]

**Response to Reviewers**

We would like to thank the Academic Editor and the reviewers for the insightful and constructive comments on our manuscript, “Prompt architecture induces methodological artifacts in large language models.” We have done our best to address all of the points raised, as detailed in this letter.

**Academic Editor:**

Thank you for the opportunity to revise our manuscript and for providing a clear roadmap forward. Below we respond to each of your requests:

1. We have ensured that the manuscript meets the PLOS ONE style requirements.
2. All data and code are and will remain publicly available on ResearchBox: https://researchbox.org/2826&PEER_REVIEW_passcode=USUUIK. In accordance with PLOS ONE’s policy, we will make everything freely accessible if our manuscript is accepted for publication.
3. In the previous version we mislabeled Figure 3 as Figure 2. We apologize for the confusion and have corrected this typo.
4. We removed supplementary figures and tables from the main text and uploaded them separately as Supplementary Information. Following your request, each Supporting Information file has a legend listed in the manuscript after the references list.

Below, we also provide a summary of the main changes we have made in response to the reviewer’s comments:

- We have incorporated references from medicine and explained how they relate to our findings.
- We have replicated our results with an open-source model, Llama-3.1.
- We have incorporated references that study other types of biases of large language models and explained how they relate to our research.
- We have further explored the pros and cons of the aggregation approach which we propose as a way to reduce methodological artifacts.
- We have polished the writing throughout and hired a professional copy editor.

**Reviewer 1:**

We appreciate your insightful comments, which were essential in guiding our revision effort. Below we quote your concerns and then summarize the steps we took to address each one.

1. “Various detailed studies in the field of Medicine have utilized multiple-choice questions, particularly in the format of A, B, and C. These studies often use complex clinical cases from sources such as the New England Journal of Medicine (NEJM) Clinicopathological Conference (CPC). With detailed reasoning, large language models (LLMs) predict the answers. These predictions, when justified, are correct answers and not biases. Any comments?”

Thank you for pointing out a very important context where our findings may or may not generalize. Indeed, there are papers that find that LLMs can be quite accurate for multiple-choice questions in medicine (Harsha et al., 2023; McDuff et al., 2023; Singhal et al., 2023). However, early evidence suggests that LLM responses might also be affected by prompt architecture in the medical context. For example, Hager et al. (2024) find that the order of options in multiple-choice questions affects LLM’s answers in clinical decision making, and Arroyo et al. (2024) find that the performance of LLMs on tasks such as mortality prediction vary based on slight differences in the wording of the instructions. Therefore, we believe our findings are also relevant to the medical domain, which we now cite thanks to your input.

2. “The sentence 'Although we find that asking for justification reduces response order and label bias, it is possible that it could exacerbate other biases we did not measure'. These other potential biases can be discussed further.”

Thank you for your suggestion to further elaborate on this interesting possibility. We include a discussion of the possible biases induced by asking for justification on page 12, lines 199-204 (following “However, research also finds that asking for reasons can bias human decision makers in other ways”).

“*For example, asking for justification leads people to select options that contain features that are easier to articulate, even if these options are less preferred (e.g., a humorous poster vs. an abstract, aesthetic one) (37). Perhaps asking an LLM to justify its selection of a job candidate or image could bias it toward decisions that are easier to articulate, such as the candidate with a higher GPA or an image with more discernible components*.”

3. “The sentence 'However, the bias appears to be less severe in GPT-3 compared to GPT-4, with 53.2% vs. 63.21% of the cases, respectively'. This important finding should be mentioned in the main manuscript, not only in the supplemental data.”

We now mention this finding in the main manuscript on page 21, lines 370-373. We further reference the finding in the abstract.

4. “Due to the proprietary nature of GPT models, there is growing interest in open-source LLMs. Is it possible to test the hypothesis with other open-source models like LLaMA-3?”

Thank you for encouraging us to replicate our effect using an open-source LLM. Following your suggestion and R2’s, we replicated both studies using Llama 3.1. We mention these replication in the abstract and on page 13, lines 232-237 and page 16, lines 301-306 of the manuscript, and report the replications in Supplements D and F. Overall, the results support our proposition that prompt architecture induces methodological bias: we document both response-order and label effects using Llama 3.1, although GPT-4 and Llama 3.1 sometimes differ in the exact the nature of these biases (e.g., Llama 3.1 exhibits stronger symbol label bias than GPT-4). This suggests that although the presence of prompt architecture effects is robust to different models and versions, the specific impact of different features of prompt architecture is subject to change.

“*We replicate these results using Llama 3.1 (see Supplemental Information D). Similar to GPT-4, Llama 3.1 exhibits response-order bias and label bias, and these biases are moderated by label type, framing, and justification, though not always in the same way as in GPT-4. Here, the prompt that yields the most bias is when sets are labeled #, %, * and Llama 3.1 is asked which set is closer. In this setup, Llama 3.1 selected % (which also happens to be the first response) in a remarkable 99.44% of the cases..*”

*“As in Study 1, we replicate this study design using Llama 3.1 (see Supplemental Information F) and again find no evidence that the intervention worked. Instead, indicating that the order and labels were chosen or randomized flipped and exacerbated the response-order effect (among those prompts, Llama 3.1 was significantly more likely to select the second item). This further underscores that although the presence of prompt architecture effects is predictable, the specific impacts are unpredictable.”*

5. Lastly, thank you for directing our attention to an incorrect reference. We have fixed this reference. Note, it is a dictionary webpage from Oxford Reference:

Oxford Reference [Internet]. Statistical Artefact. Available from: https://www.oxfordreference.com/display/10.1093/oi/authority.20110803095426317

**Reviewer 2:**

We appreciate your thoughtful comments and helpful feedback. We have implemented your suggestions and believe that the paper is much stronger as a result. Below we quote your concerns and then summarize the steps we took to address each one.

1. “Firstly, the task itself needs to be more clearly defined. The authors should specify the exact types of labels they expect to encounter and analyze. This clarity will provide a better framework for understanding the scope and objectives of their study.”

Thank you for the opportunity to clarify the scope and objectives of our study. First, to clarify, labels in our context are *not* related to the topic of data labeling in machine learning. Instead, we are interested in the order (e.g., whether an option is presented in the first, second or third position) and labels (e.g., whether an option is labeled as “A” or “B” or “C”) assigned to the various response options in multiple choice evaluation tasks. In our context, labels are selected by the user engaging with the LLM and they should theoretically have no effect on the answer provided by the LLM because they are not a reflection of the data. We have clarified this context in the example we provide in the introduction as well as the use cases on Page 4 lines 59-65.

*“For example, managers (researchers) interested in replacing their workforce (human subjects) with generative AI may ask GPT to evaluate the similarity between different sets of items. They also need to decide how to label each set in the prompts they submit to GPT, the order in which to list the sets, and how to word the exact task. For example, a user may decide to write the prompt: “Is Set B or Set C closer to Set A?” Evaluative tasks such as these, with multiple choices that are ordered and labeled, span many different LLM use cases, including diagnoses (9,11), opinion polls and surveys (2,3), and social science experiments (5,7,8).”*

We also include examples of other labels that LLMs might encounter on pages 4-5, lines 75-77:

*“In other words, without taking this form of bias into account, a user may erroneously conclude that set A is closer to set B, candidate 1 is more qualified than candidate 2, or diagnosis I is more likely than diagnosis II, simply because of the architecture of the prompt.”*

2. “Secondly, the language throughout the paper requires some refinement. At certain points, the writing becomes too informal, which detracts from the scholarly tone of the work. A more formal and consistent style would improve the overall readability and professionalism of the paper.”

Thank you for pointing out that, at times, our writing came across as too informal. We have updated our writing throughout the paper in order to better reflect a scholarly tone. For example:

- We replaced the colloquial word “mimicking” with a more accepted term “mirroring”
- We reworded “Is it possible that a less familiar framing leads GPT to process in a way that appears more thorough, just as the request for justification?” to “Future research may test whether a less familiar framing leads LLMs to process in a way that appears more thorough, similarly to a request for justification.”
- We removed the reference suggesting that GPT might get “lazier” during holidays, as this was not from an academic source.

We also hired a professional copyeditor to further improve the paper’s writing.

3. “Additionally, the authors focus exclusively on GPT-4. This focus raises concerns about potential bias towards proprietary models. It would be beneficial to include a comparison with open-source large language models (LLMs) such as LLaMA or Mistral to provide a more balanced analysis.”

Thank you for encouraging us to replicate our effect using an open-source LLM. Following your suggestion and R1’s, we replicated both studies using Llama 3.1. We mention these replication in the abstract and on page 13, lines 232-237 and page 16, lines 301-306 of the manuscript, and, and report the replications in Supplements D and F. Overall, the results support our proposition that prompt architecture induces methodological bias: we document both response order and label effects using Llama 3.1, although GPT-4 and Llama 3.1 sometimes differ in the exact the nature of these biases (e.g., Llama 3.1 exhibits stronger symbol label bias than GPT-4). This suggests that although the presence of prompt architecture effects is robust to different models and versions, the specific impact of different features of prompt architecture is subject to change.

“*We replicate these results using Llama 3.1 (see Supplemental Information D). Similar to GPT-4, Llama 3.1 exhibits response-order bias and label bias, and these biases are moderated by label type, framing, and justification, though not always in the same way as in GPT-4. Here, the prompt that yields the most bias is when sets are labeled #, %, * and Llama 3.1 is asked which set is closer. In this setup, Llama 3.1 selected % (which also happens to be the first response) in a remarkable 99.44% of the cases.*”

*“As in Study 1, we replicate this study design using Llama 3.1 (see Supplemental Information F) and again find no evidence that the intervention worked. Instead, indicating that the order and labels were chosen or randomized flipped and exacerbated the response-order effect (among those prompts, Llama 3.1 was significantly more likely to select the second item). This further underscores that although the presence of prompt architecture effects is predictable, the specific impacts are unpredictable.”*

4. “The definition of bias within the context of the study is another critical area that needs attention. The authors should refer to relevant definitions and frameworks, such as those discussed in the paper available at [IEEE Xplore](https://ieeexplore.ieee.org/abstract/document/10538997), to establish a definition and solid theoretical foundation for their analysis. “

Thank you for encouraging us to define bias in the context of our study and for the excellent reference. We now contrast how bias has been defined and explored in prior literature with our definition to (a) better couch our findings in the extant literature and (b) clarify our contribution. In our context, bias is defined as a form of statistical bias, specifically methodological artifacts. Our work on statistical bias complements the very important extant work in LLMs on social bias, which studies “the predisposition or inclination toward a particular group (based on gender or race)... that can unduly influence the representation or treatment of particular groups.” (Raza et al., 2024) These edits are reflected in the text on pages 3-4, lines 47-55:

“*In this article, we complement work that defines bias as “the predisposition or inclination toward a particular group (based on gender or race), often rooted in social stereotypes, that can unduly influence the representation or treatment of particular groups” (16) by focusing on methodological artifacts, or biases due to the specificities of a given task design. Methodological artifacts are a form of statistical bias, as they are systematic errors that lead to conclusions that are partly “a result of the particular research technique employed, rather than an accurate representation of the world” (18). Using a choice architecture framework, we examine how arbitrary associations embedded in the rich language data used to train LLMs may induce statistical bias (and, therefore, errors) in output.*”

Defining our bias as a form of statistical bias provides a foundation for our design and analysis, as we delineate on page 7, lines 134-138:

*“This is a full factorial design in which each set has one instance of each order and label combination, which allows us to test for statistical bias because we experimentally manipulate order and label independent of the actual content of the set. As a result, any impact of order and/or label is definitionally biased and does not reflect a real difference between sets*.”

We believe the updated manuscript provides a fuller picture of how statistical bias can manifest in LLM output, clarifies our contribution, and grounds our work by providing a theoretical foundation for analysis.

5. “The literature review needs to be updated to reflect the latest research and developments in the field. This update will ensure that the study is situated within the current academic discourse and acknowledges recent advancements and discussions.”

Thank you for ensuring our literature review of this fast-moving field is up to date. We have updated the status of our references, surveyed recent additions to the academic discourse and identified new papers to include in our review.

In particular, we added the following references that have studies LLM biases of a social nature:

- Raza S, Bamgbose O, Chatrath V, Ghuge S, Sidyakin Y, Muaad AY. Unlocking bias detection: leveraging transformer-based models for content analysis [Internet]. arXiv; 2024 [cited 2024]. Available from: http://arxiv.org/abs/2310.00347
- Raza S, Rahman M, Zhang MR. BEADs: bias evaluation across domains [Internet]. arXiv; 2024 [cited 2024]. Available from: http://arxiv.org/abs/2406.04220
- Zack T, Lehman E, Suzgun M, Rodriguez JA, Celi LA, Gichoya J, et al. Assessing the potential of GPT-4 to perpetuate racial and gender biases in health care: a model evaluation study. The Lancet Digital Health. 2024;6(1):e12–22.

We have added the following reference relevant to our findings, which was posted after our initial submission: Reif Y, Schwartz R. Beyond Performance: Quantifying and mitigating label bias in LLMs [Internet]. arXiv; 2024 [cited 2024]. Available from: http://arxiv.org/abs/2405.02743

Finally, following some of R1’s suggestions, we have added references that explore the use of LLM in medical decision making:

- Arroyo AMC, Munnangi M, Sun J, Zhang KYC, McInerney DJ, Wallace BC, et al. Open (Clinical) LLMs are sensitive to instruction phrasings [Internet]. arXiv; 2024 [cited 20249]. Available from: http://arxiv.org/abs/2407.09429.
- Hager P, Jungmann F, Holland R, Bhagat K, Hubrecht I, Knauer M, et al. Evaluation and mitigation of the limitations of large language models in clinical decision-making. Nat Med. 2024
- McDuff D, Schaekermann M, Tu T, Palepu A, Wang A, Garrison J, et al. Towards Accurate Differential Diagnosis with Large Language Models [Internet]. arXiv; 2023 [cited 2024]. Available from: http://arxiv.org/abs/2312.00164
- Nori H, King N, McKinney SM, Carignan D, Horvitz E. Capabilities of GPT-4 on medical challenge problems [Internet]. arXiv; 2023 [cited 2024]. Available from: http://arxiv.org/abs/2303.13375
- Singhal K, Azizi S, Tu T, Mahdavi SS, Wei J, Chung HW, et al. Large language models encode clinical knowledge. Nature. 2023;620(7972):172–80.

6. “Clarification is also needed on whether the labeling process employed in the study is stochastic. Understanding the nature of the labeling process is essential for assessing the reliability and consistency of the generated labels.”

Thank you for encouraging us to clarify how we evaluate the effect of labels in our study (keeping in mind that labels in our context are *not* related to the topic of data labeling in machine learning). In order to test the effect of an arbitrary label (e.g., a set being labeled A or B), we experimentally manipulate the labels used for each set. This allows us to hold everything constant except the labels being used to designate the set. For example, the same triplet labeled with letters would be presented four different ways (*A*. Set 1, *B*. Set 2, *C*. Set 3; *A*. Set 1, *C*. Set 2, *B*. Set 3; *A*. Set 1, *B*. Set 3, *C*. Set 2; *A*. Set 1, *C*. Set 3, *B*. Set 2) in our study.

We clarify the labeling process used in our experiment on page 7, lines 134-136.

*“This is a full factorial design in which each set has one instance of each order and label combination, which allows us to test for statistical bias because we experimentally manipulate order and label independent of the actual content of the set.”*

We have also clarified that although the labeling and order of options in a single prompt in our studies was randomly assigned using a full-factorial design, the labeling process in the real world may problematically reflect a user's expectation or desire. We reflect on this possibility on page 17, lines 320-323.

*“Moreover, in practice, order and labels may not be applied randomly. For example, users may list their personal favorite option first. Thus, the results we report suggest that LLMs may inadvertently serve as a confirmation bias tool rather than an unbiased adviser.”*

7. “The authors should provide a thorough discussion of the pros and cons of their approach. This analysis will help readers understand the strengths and limitations of the method and its potential implications for future research and applications.”

In the updated manuscript, we now explicitly suggest aggregation across prompts as a method to reduce the statistical bias induced by prompt architecture. The pro of this approach is that it fully removes bias while also increasing accuracy. The cons are that it might be time and resource-intensive to construct a full factorial design of prompts to aggregate. We detail this on page 18-21, starting on line 342:

*“We find that the extent of bias varies across prompts, with the most biased prompts selecting the arbitrary Set 2 100% of the time across all triplets and categories (see Fig. 4). The specific prompts that demonstrate the least bias (e.g., approach 50% selection of Set 2) are different across the two models, suggesting that there is no ideal “unbiased” prompt. Instead, we suggest aggregating across different prompt types, that is, simply using the majority answer across prompt variations: for a particular triplet, across the 32 prompt formats we count the number of times the model indicated that Set 2 was closer and the number of times it indicated that Set 3 was closer, and we use the majority answer as the final answer. We find that this simple approach eliminates bias: Set 2 is selected 50% of the time when aggregating across all 32 prompts for both GPT-4 (50.01%) and Llama 3.1 (50.06%).*

*Note that eliminating bias does not necessarily improve accuracy. For example, a coin flip would produce an unbiased but inaccurate answer. To ensure that our intervention did not compromise accuracy, we ran an experiment identical to Study 1 but with single-word items and used word embeddings to calculate a noisy proxy for similarity and, in turn, GPT-4’s accuracy when making similarity judgments. Our results suggest that aggregating reduces bias while increasing accuracy (see Supplemental Information G). Thus, the benefit of aggregation is that it reduces bias while improving accuracy. The drawback is that it requires identifying the elements of prompt architecture that might bias an LLM’s response and creating a full factorial set of prompts to address this bias, and doing so could be time and resource intensive. In cases in which this is too daunting, we recommend, at minimum, balancing order and labeling.”*

8. “From what I understand, the authors aim to mitigate the biases in GPT-generated labels by proposing a more reliable approach. To enhance the practical value of their study, they should include an algorithm presented in pseudocode. This inclusion would provide a clear, actionable method for other researchers and practitioners to implement and build upon their work.”

We would like to clarify that labels in our context are not generated by GPT. In our experiments, labels are varied experimentally by us. In the type of real-world applications that we study, labels are assigned by the user asking a multiple-choice question to the LLM.

We realize that aggregation was not defined clearly enough in the previous draft and apologize for the confusion. Given it is a very simple approach, we describe it in words rather than in algorithmic form, on page 18-19, lines 345-349:

*“Instead, we suggest aggregating across different prompt types, that is, simply using the majority answer across prompt variations: for a particular triplet, across the 32 prompt formats we count the number of times the model indicated that Set 2 was closer and the number of times it indicated that Set 3 was closer, and we use the majority answer as the final answer.”*
