# Supplementary material for: Prompt architecture induces methodological artifacts in large language models
Source: PLoS One. 2025 Apr 28;20(4):e0319159. doi: 10.1371/journal.pone.0319159 (PMC12036937; doi:10.1371/journal.pone.0319159)
Supplement: Attachment — Submitted filename: Response to Reviewers.docx [file pone.0319159.s011.docx]

**Response to Reviewers**

We would like to thank the Academic Editor and the reviewers for their time and feedback on our manuscript, “Prompt architecture induces methodological artifacts in large language models.” We have done our best to address the points raised by reviewer 3, as detailed in this letter.

**Reviewer 3:**

Thank you for stepping in and taking the time to carefully read our revision and give us detailed comments on how to improve the paper! Below, we respond to each of your line-by-line comments.

1. 38: Consider rephrasing “virtually unlimited memory and processing abilities” to avoid overstating LLMs computing power. While it is true that LLMs do not experience fatigue or require rest like humans, they are subject to memory constraints (e.g., character limits in prompts).

We have updated our writing to more accurately reflect LLM memory and processing constraints on line 38:

“*LLMs have extensive memory and processing abilities, are presumably not influenced by emotions or moods, and never lack motivation*.”

2. 53: Applying the choice architecture framework is very appropriate. However, additional citations beyond the foundational work by Thaler and Sunstein (2009) would strengthen the paper. Possible sources include:

Johnson, E. J., Hershey, J., Meszaros, J., & Kunreuther, H. (1993). Framing, probability distortions, and insurance decisions. Journal of Risk and Uncertainty 7, 35–51. <https://doi.org/10.1007/BF01065313>

Mrkva, K., Posner, N. A., Reeck, C., & Johnson, E. J. (2021). Do nudges reduce disparities? Choice architecture compensates for low consumer knowledge. Journal of Marketing, 85(4), 67–84. <https://doi.org/10.1177/0022242921993186>

Thaler, Richard H. and Sunstein, Cass R. and Balz, John P., Choice Architecture (April 2, 2010).

http://doi.org/10.2139/ssrn.1583509

We updated our references to include the additional references you suggested.

3. 69: The initial clarification that “justification” equates to “asking for reasons” is helpful. Afterward, maintaining consistent terminology by using justification aligns better with the choice architecture framework.

We have updated our writing to consistently refer to the “asking for reasons” element as “justification.”

4. 120: While this similarity task may be standard in psychology, the rationale for selecting it here is unclear. Could a more complex task (e.g., categorization) or simpler one (e.g., recognition) have provided additional insights relevant to the study?

We included an additional paragraph in the General Discussion starting on line 429 discussing the generalization of our tasks to more or less complex tasks. We quote the most relevant part of the text below.

*“Our empirical context involved both simple and complex similarity tasks and formally worded prompts, which represents only a subset of the diverse types of prompts users might provide to LLMs. This raises the question of how our findings generalize to other contexts, such as tasks that vary in complexity beyond the similarity tasks in our studies or prompts that are phrased less formally than our prompts. Prior research with human participants provides a few hypotheses. First, human respondents are more likely to be influenced by choice architecture during complex tasks because these tasks involve more in-the-moment response construction (23, 43). Thus, our observed effects of prompt architecture may exacerbate for more complex tasks and attenuate for simpler tasks compared to the similarity task used in our studies. However, of note, we observed effects of prompt architecture for both simple (i.e., one-item stimuli) and complex (i.e., five-item stimuli) similarity tasks.”*

5. 126–141: The full-factorial design described here is both clever and effective. Presenting the information as a summary table could improve clarity. For instance:

Thank you for this nice suggestion! We have added the table to the paper on line 164.

6. 147–148: The structured prompt used in the study is well-suited for research but may not reflect typical user behavior. Users often input shorthand, unstructured queries, such as:
   “is set b or set c closer to set a: egypt us germany australia france. set b: brazil japan south africa spain. set c: canada uk china india argentina”

Has GPT-4’s performance with less structured inputs been evaluated? While the authors note that GPT-4 will eventually be succeeded by future models, acknowledging that user prompts are often informal and variable could add practical depth to the analysis.

We agree that the formality of prompts is an interesting element of prompt architecture that could affect LLM responses. We now acknowledge that our prompts are formal and reflect in the General Discussion starting on line 429 on how prompt informality may moderate our effects. We quote the most relevant text below.

*“Second, research suggests that informality during communication, such as text abbreviations, can signal low effort and, as a result, decrease responsiveness (44). If this pattern exists in the training data, LLMs may be less responsive (e.g., provide less thorough justification) when the prompt contains more informal language usage. We encourage future research to explore the generalizability of our findings, including how complexity moderates the effects of prompt architecture and how additional architectural elements beyond the ones examined in this paper, such as prompt formality, may influence LLM responses.”*

7. 184–186: The figures are informative, but it might help if Figures B and C are grouped together, as they both illustrate label bias. Additionally, consider swapping the two framing bars in Figure A with the two label bars. This adjustment would align the visual organization with Figures B and C

We grouped figure B and C together on the bottom panel and swapped the two framing bars in Figure A with the two label bars. Thank you for these suggestions—we agree this improves the visualization of our findings.

8. 192–205: The parallels between human and LLM decision-making are fascinating but could be misleading. The phrasing might suggest that LLMs have emotions or motivations. Emphasizing that these changes reflect structural patterns, not emotions, would clarify the distinction.

We clarified this distinction by adding the following line on 228-230:

*“Although LLMs do not experience emotions or motivation, structural patterns in the training set could lead to similar results.”*

9. 225: Consider replacing “irrationally” with “predictably,” as these biases follow rational heuristics for efficiency. Both humans and LLMs are acting consistently based on context.

We removed the word “irrationally.”

10. 281: Including the percentage of failures would contextualize the impact of 489 errors. Additionally, the reported figure of 64,800 observations seems to include an extra “0.”

We included the failure rate and removed the extra 0. Thank you for catching that!

11. 358–365 & 367–376: These points are significant and belong in the methods section rather than the discussion, as they are not new findings but validation steps. The discussion should focus on interpreting results rather than supplying new information. Also, explicitly state that Word2Vec was used for word embeddings, as it is currently only specified in the supplementary materials.

We agree with you that both the GPT-3 replication and the accuracy measure are significant additions. Following your recommendation, we now mention our GPT-3 replication in the methods section (following study 1 results, starting on line 263). However, after much deliberation we opted to keep the accuracy measure in the Discussion section because it seems to us that its calculation is only relevant to exploring the potential benefit of aggregation and does not speak directly to our experimental findings.

We also now explicitly mention that we use Word2Vec for word embeddings on line 411.

12. 376–378: Referring back to lines 109–111:
    The recommendation to aggregate across different prompts is valuable, but it raises practical questions. Specifically:

Is the final recommendation to use a full factorial search query? If so, this approach may be impractical for many users.
If not a full factorial search, could the authors suggest a reasonable number of prompts that would sufficiently reduce bias?

We agree with your analysis that running a full factorial design is impractical for many users, at least for each and every prompt they write. We now acknowledge this limitation on line 113:

*“Although developing a full factorial design each time a user prompts a LLM may be impractical, the benefits likely outweigh the costs for decisions where bias is particularly harmful, such as in hiring or diagnoses. In addition, such factorial design may be implemented automatically (and potentially in a way that is transparent to the end user) by developing simple applications that generate the design and run it on the API.”*

Although it would be helpful to have a rule of thumb regarding the number of prompts to use to sufficiently reduce bias (while improving accuracy), this isn’t feasible because prompt architecture effects are definitionally contextual—any given prompt will vary on the elements a user should consider. The goal of this paper is to provide a choice architecture framework to help users identify where bias may lurk.

13. **Comments on Supplementary Material B:**

There appears to be a typo: “If the prompt uses letters instead of symbols: replace A with #, B with %, C with .” It seems “letters” and “symbols” need to be swapped.

Fixed! Thanks!

14. Additionally, the third technique described—swapping sets while maintaining labels—is a key aspect of prompt generation and should be referenced in the main text, not just the supplementary materials.

Thank you. We now explicitly mention in the main text starting on line 148 that the order manipulation consists of swapping the second and third sets*.*

“*Second, we independently vary order by swapping the second and third labels (A, B, C vs. A, C, B or #, %, * vs. #, *, %) and the second and third sets (123 or 132).”*
